# Impact of Phosphorylation at Various Sites on the Active Pocket of Human Ferrochelatase: Insights from Molecular Dynamics Simulations

**DOI:** 10.3390/ijms25126360

**Published:** 2024-06-08

**Authors:** Mingshan Guo, Yuhong Lin, Chibuike David Obi, Peng Zhao, Harry A. Dailey, Amy E. Medlock, Yong Shen

**Affiliations:** 1 School of Chemistry, IGCME, Sun Yat-sen University, Guangzhou 510006, China; 2 Department of Biochemistry and Molecular Biology, University of Georgia, Athens, GA 30602, USA; chibuike.obi@cchmc.org (C.D.O.); hdailey@uga.edu (H.A.D.); medlock@uga.edu (A.E.M.); 3 Complex Carbohydrate Research Center, University of Georgia, Athens, GA 30602, USA; pengzhao@ccrc.uga.edu; 4 Augusta University/University of Georgia Medical Partnership, Athens, GA 30602, USA

**Keywords:** human ferrochelatase, phosphorylation, molecular dynamics

## Abstract

Ferrochelatase (FECH) is the terminal enzyme in human heme biosynthesis, catalyzing the insertion of ferrous iron into protoporphyrin IX (PPIX) to form protoheme IX (Heme). Phosphorylation increases the activity of FECH, and it has been confirmed that the activity of FECH phosphorylated at T116 increases. However, it remains unclear whether the T116 site and other potential phosphorylation modification sites collaboratively regulate the activity of FECH. In this study, we identified a new phosphorylation site, T218, and explored the allosteric effects of unphosphorylated (UP), PT116, PT218, and PT116 + PT218 states on FECH in the presence and absence of substrates (PPIX and Heme) using molecular dynamics (MD) simulations. Binding free energies were evaluated with the MM/PBSA method. Our findings indicate that the PT116 + PT218 state exhibits the lowest binding free energy with PPIX, suggesting the strongest binding affinity. Additionally, this state showed a higher binding free energy with Heme compared to UP, which facilitates Heme release. Moreover, employing multiple analysis methods, including free energy landscape (FEL), principal component analysis (PCA), dynamic cross-correlation matrix (DCCM), and hydrogen bond interaction analysis, we demonstrated that phosphorylation significantly affects the dynamic behavior and binding patterns of substrates to FECH. Insights from this study provide valuable theoretical guidance for treating conditions related to disrupted heme metabolism, such as various porphyrias and iron-related disorders.

## 1. Introduction

Protoheme IX (Heme), an essential cofactor of a variety of proteins, not only participates in numerous biochemical processes, such as gas binding and transport, peroxidase catalysis, and one electron transfer reactions, but also functions as a regulator of various essential cellular processes, including gas sensing, microRNA splicing, protein degradation, and the circadian clock [1,2,3,4,5,6,7]. However, due to the chemical reactivity and destructive nature of free heme towards cellular structures, precise regulation of its synthesis and degradation is essential.

**Figure 1:**
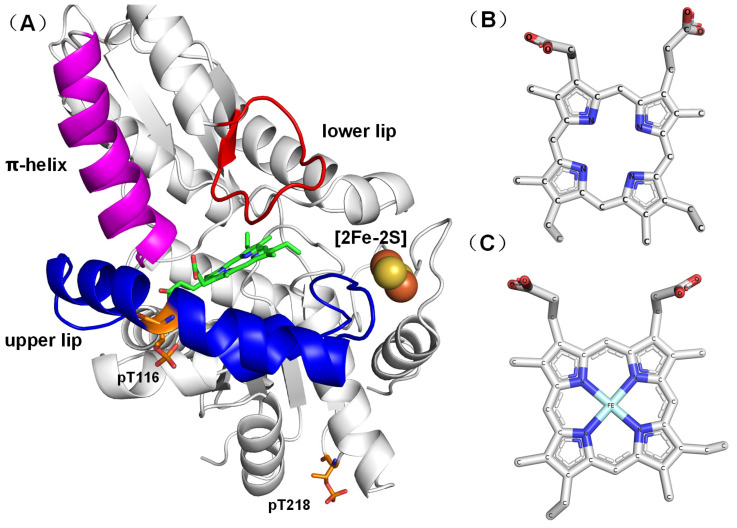
Structure of FECH-PPIX (PT116 + PT218) generated from the E343K variant structure (PDB 2QD1) [11]. (**A**) Different structural parts are shown, including residues 340–349, which comprise the π-helix (purple); residues 90–130, constituting the upper lip of the active site (blue); residues 300–311, forming the lower lip region (red); the [2Fe-2S] cluster (yellow and orange); and PT116 and PT218 (orange). Substrate (PPIX) in a ball-and-stick model (green). (**B**) Stick representation of PPIX. (**C**) Stick representation of heme.

Significant research has been conducted to investigate and understand the complex intricacies of heme biosynthesis [8]. The initial step in heme synthesis is the formation of 5-aminolevulinic acid (ALA), followed by a number of condensation reactions to form the first cyclic tetrapyrrole, uroporphyrinogen III. The pathway from ALA to uroporphyrinogen III involves three enzymes that are highly conserved across nature. There are three distinct routes to heme from uroporphyrinogen III in prokaryotes: the protoporphyrin-dependent pathway, the coproporphyrin-dependent pathway, and the siroheme pathway [9]. Only the protoporphyrin-dependent pathway exists in eukaryotes. In this pathway, the terminal step is the insertion of ferrous iron into protoporphyrin IX (PPIX) to form heme. This step is catalyzed by the mitochondrially located enzyme ferrochelatase (FECH) [10] (Figure 1A).

Mutations in the *FECH* gene, which lead to a reduction in FECH activity, result in a disorder known as erythropoietic protoporphyria (EPP) [12,13]. In individuals with EPP, the inefficient conversion of PPIX into heme results in the accumulation of PPIX, a phototoxic compound that can cause severe dermatologic pain and sunlight sensitivity [13,14,15]. To date, more than 160 EPP-causing mutations in FECH have been reported in the Human Gene Mutation Database (URL https://doi.org/www.hgmd.cf.ac.uk (accessed on 28 February 2024)). While extensive studies on human FECH exist and provide a good picture of its function [11,16,17], the molecular basis for enzyme dysfunction has not been identified for all reported variants. Therefore, comparing wild-type with mutant FECHs is crucial for the development of effective therapeutic strategies to manage and treat EPP.

There are significant differences between the metallized and nonmetallized porphyrin structures in human FECH. Specifically, the R115L mutant (PDB ID 2HRC) [16] demonstrates an open conformation whereas the E343K mutant, which is bound to PPIX (PDB ID 2QD1) [11], shows a closed conformation. In this state, PPIX is fixed at the active site, facilitating the insertion of ferrous ions into PPIX. Additionally, the F110A mutant, when associated with the product (Heme), is demonstrated in a release conformation (PDB ID 2QD2) [11], implying that FECH releases heme following the insertion of the ferrous ions into PPIX. The sequence of conformational changes in the catalytic cycle of FECH proceeds from open to closed to release, before returning to the open state for the initiation of a new cycle. These conformational transitions involve the π-helix (amino acid residues 340–349) (Figure 1A) being unwound in the open conformation. In the closed and release conformations, changes in the state of the π-helix, the upper lip region (amino acid residues 90–130), and the lower lip region (amino acid residues 300–311) along with shifts in the position and orientation of PPIX, trigger a series of conformational changes. The upper lip region (amino acid residues 90–130) is known for the pronounced mobility during PPIX binding [11] (Figure 1A).

Posttranslational modifications (PTMs), such as phosphorylation, have been identified as key elements crucial for regulating protein function [18,19]. FECH, akin to numerous other proteins, has been shown to undergo phosphorylation, a process in which phosphate groups are covalently attached to specific amino acid residues by protein kinases. Research has demonstrated that the phosphorylation of FECH by protein kinases, such as protein kinase C (PKC) and protein kinase A (PKA), can significantly modulate FECH enzyme activity [20,21,22]. PKA-induced phosphorylation of T116 (PT116 in Figure 1A) triggers a conformational change, moving the bulky phosphorylated T116 away from H86 on another α-helix. This conformational alteration has been proposed to expand the active site pocket, facilitating more efficient movement of the active site lips during catalysis and enhancing catalytic efficiency [20]. Further investigation is required to elucidate the possible impact of this PTM on FECH activity.

In this study, we investigated additional sites of FECH phosphorylation and employed molecular dynamics (MD) simulations to explore the intricate relationship between phosphorylation and FECH activity. These simulations serve as a valuable tool for examining the structural changes induced by phosphorylation, as well as the interactions between FECH and substrates of heme synthesis, such as PPIX and ferrous iron. By shedding light on the regulatory mechanisms governing heme production, this research has significant implications for the development of novel therapeutic strategies targeting heme-related disorders. Modulation of FECH activity through the regulation of PTMs, specifically phosphorylation at T116 and T218 (PT116 and PT218), may offer new avenues for treating conditions associated with disrupted heme metabolism, including various porphyrias and iron-related disorders.

## 2. Results and Discussion

### 2.1. Crystal Structure

**Figure 2:**
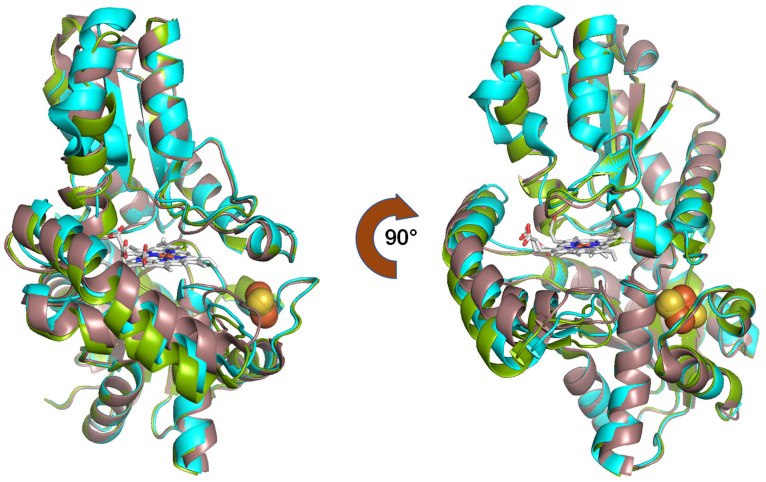
Crystal structures show structural differences in the conformations of the FECH complexes. R115L variant is shown in green, E343K variant in brown, and F110A variant in cyan.

As an initial step towards comparing the structure of FECH, we initially overlaid the available crystal structures of the complexes. The structures were aligned based on the positions of FECH Cα atoms (Figure 2). The E343K variant exhibited a “closed” conformation, while the R115L variant showed an “open” conformation. These conformations potentially regulate PPIX binding and heme release. The E343K variant increased the enzyme’s affinity for PPIX without causing irreversible binding. In the F110A variant, the π-helix was “unwound” due to heme binding [11]. The initial structure reported for human FECH was the R115L variant, which has catalytic properties nearly identical to those of the wild-type recombinant enzyme [16]. This revealed significant structural differences among the three conformations.

The mutants R115L, E343K, and F110A, located at the active site of the FECH enzyme, significantly influenced the enzyme’s function and stability. Additionally, the mutants solved by X-ray diffraction may occupy different minimum energy states compared to wild-type enzymes. Consequently, these different energetic states may induce conformational changes, including subtle variations such as dihedral angle shifts in adjacent residues. However, because data on the crystallization of the wild-type protein were unavailable currently, we could not include them in our analysis. In this research, all the mutated residues present in the crystal structures were changed back to the wild-type sequence. And our results indicated that long-term molecular dynamics simulations enable an accurate investigation of the wild-type enzyme’s behavior. A 1 μs molecular dynamics simulation provided sufficient sampling to reflect the intrinsic dynamics and conformational flexibility of the wild-type protein, ensuring a more accurate representation of its natural state [23,24]. With more data on the crystallization of the wild-type protein available in the future, comparative analyses including them will enhance our understanding of the FECH enzyme’s full ensemble of conformations.

### 2.2. Identification of FECH Phosphorylation Sites

To investigate the sites of FECH phosphorylation, we co-expressed His-tagged human FECH and PKA in *Escherichia coli*. Following purification, the samples were analyzed for global phosphorylation. In addition to the previously identified T116 [20], we found three other sites with significant levels of modification via phosphorylation, including T218, S370, and S416. Among all the phosphorylated residues, the T218 modification was highest, with 48–73% occupancy, as compared to the T116 site with 18–40%, S370 with 26–41%, and S416 with 3–11% for wild-type FECH and increased in T116A FECH (Supplemental Table S1).

### 2.3. Structural Analysis of Phosphorylated FECH

The root mean square deviation (RMSD) is a fundamental metric employed in MD simulations to evaluate the structural variability of systems. In this study, RMSD analysis was performed over a simulation time of 1 μs, capturing the conformational dynamics and structural changes of Cα atoms in all systems within this time interval. The stability of FECH systems was assessed by measuring the RMSD values and representing them graphically [25].

**Figure 3:**
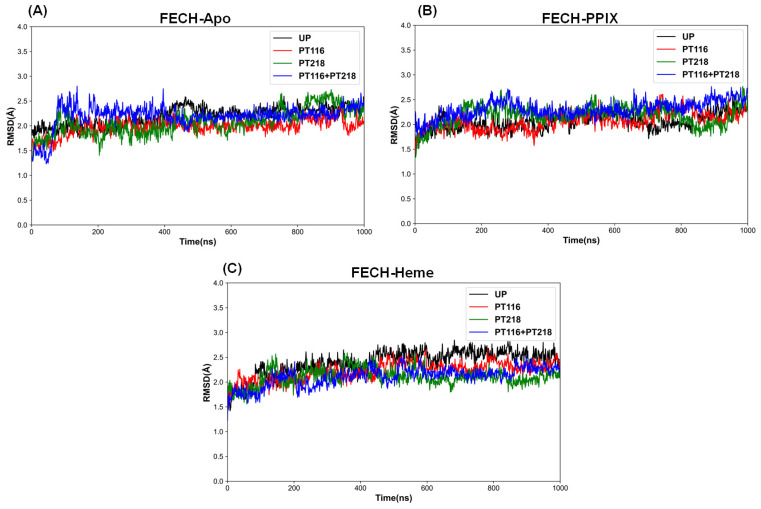
Time evolution of the RMSDS of the Cα atoms during the 1 μs simulations of all the complex systems using the initial structure as a reference. (**A**) the FECH-Apo systems; (**B**) the FECH-PPIX systems; (**C**) the FECH-Heme systems.

For the FECH-Apo systems (Figure 3A), FECH exhibited considerable stability across four phosphorylation states: unphosphorylated (UP), single-point phosphorylated (PT116 or PT218), and double-point phosphorylated (PT116 + PT218). The average RMSD values of the Cα atoms in these systems ranged from 1.90 to 2.20 Å (Table 1) during a 1 μs MD simulation. In the FECH-PPIX systems (Figure 3B), four phosphorylated states in complex with PPIX reached dynamic equilibrium at approximately 400 ns, with RMSD values mainly ranging between 2.0 and 2.30 Å (Table 1). Notably, PT218 single-point phosphorylation and PT116 + PT218 double-point phosphorylation were associated with higher RMSD values in the early simulation phase (approximately 200 ns), suggesting larger conformational shifts. However, these systems also stabilized gradually as the simulation proceeded. In the FECH-Heme systems (Figure 3C), the four phosphorylation conditions in complex with heme reached dynamic equilibrium after 500 ns of simulation, with RMSD values between 2.10 and 2.40 Å (Table 1). This finding implied that the presence of heme may induce protein dynamics.

The root mean square fluctuation (RMSF) is a crucial metric used in MD simulations to evaluate the local flexibility and dynamic behavior of atoms within a system [26,27]. This approach provides insight into the extent to which atoms deviate from their average positions over the course of the simulation trajectory. In this analysis, RMSF values were computed by measuring the displacements of Cα atoms from their mean coordinates.

**Figure 4:**
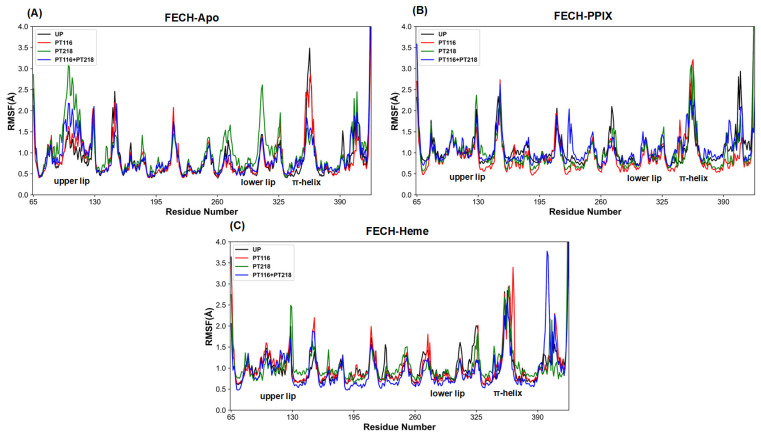
RMSFs of backbone atoms of all systems using the average structure during the 1 μs simulations. (**A**) The FECH-Apo systems; (**B**) the FECH-PPIX systems; (**C**) the FECH-Heme systems.

In the FECH-Apo systems, a significant increase in RMSF values was observed for the upper lip and lower lip regions in PT218 and PT116 + PT218 (Figure 4A), suggesting that phosphorylation of T218 may enhance dynamism in these regions, potentially leading to conformational changes in these domains. The π-helix showed higher RMSF values in UP and PT116, indicating increased dynamism in these states. In the FECH-PPIX systems (Figure 4B), the π-helix region in UP and PT116 + PT218 had lower RMSF values, indicating enhanced stability after PPIX insertion. However, for the upper lip region and lower lip region, the RMSF values remained similar across the four phosphorylation states, suggesting that different phosphorylation sites have less impact on the dynamism of these regions. In the FECH-Heme systems (Figure 4C), similar RMSF values were observed in all three regions (π-helix, upper lip region, lower lip region) across all phosphorylation states. This likely indicates that the different phosphorylation sites have almost no effect on the flexibility of the regions.

**Figure 5:**
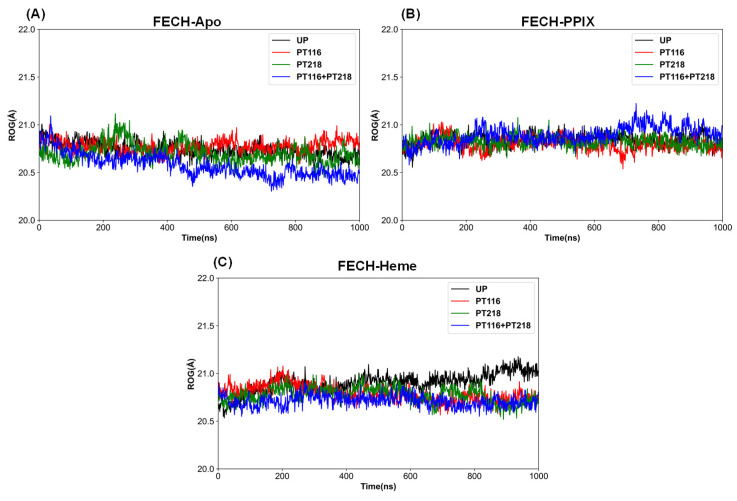
ROG of Cα atoms of all systems during the 1 μs simulations. (**A**) The FECH-Apo systems; (**B**) the FECH-PPIX systems; (**C**) the FECH-Heme systems.

The radius of gyration (Rg), the root mean square distance of parts of an object from either its center of mass or a given axis, serves as an indicator of the object’s compression behavior or compactness. This measure is also useful for assessing protein compactness [28]. In the FECH-Apo systems, the Rg values gradually decreased across the four phosphorylation states, indicating a transition from relatively loose to more compact structures. The Rg values for single phosphorylation (PT116 and PT218) were similar to the those of the unphosphorylated (UP) state, while the PT116 + PT218 state showed the lowest Rg value among the four states (Figure 5A). In the FECH-PPIX systems, there was no clear trend of structural compression or expansion; the Rg values were nearly identical in the unphosphorylated and single phosphorylation states, with the PT116 + PT218 state exhibiting a slightly larger Rg value and greater fluctuation (Figure 5B). In the FECH-Heme systems, the phosphorylated states had lower Rg values compared to the unphosphorylated state, and the Rg values in the UP state gradually increased, indicating a more loosened structure (Figure 5C).

### 2.4. Residue Interactions of the Active Pocket

**Figure 6:**
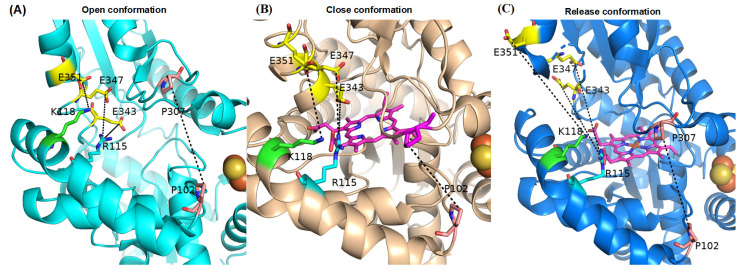
The active pocket residues of the wide-type, metallized and nonmetallized porphyrin structures. (**A**) The FECH-Apo system without substrate in the open conformation; (**B**) the FECH-PPIX system bound to PPIX in closed conformation; (**C**) the FECH-Heme system with heme in the release conformation.

The active pocket of FECH is composed of multiple charged residues distributed across the upper lip region, the lower lip region, and the π-helix, forming a hydrogen-bonding network that confers stability to FECH in the open conformation (Figure 6A) [29]. Upon PPIX binding, the active pocket undergoes closure, resulting in the disruption of electrostatic interactions and facilitating the transition to the closed conformation (Figure 6B) [11]. The insertion of iron into the active site triggers the conformational transformation of FECH into the release conformation (Figure 6C) [11]. The distance between the upper lip region and the lower lip region (P107–P307), the interactions between the upper lip region and the π-helix including R115–E343, R115–E347, and K118–E351, and time evolutions of the interaction distances between selected residue pairs during the 1000 ns MD simulations in FECH-Apo systems, FECH-PPIX systems and FECH-Heme systems are shown in Figures S1–S3.

In the FECH-Apo system without phosphorylation, the conformation was open. After phosphorylation, the distance between R115 and E343 increased, whereas the distance between K118 and E351 decreased, indicating a consistent trend (Table 2). Notably, under the PT116 condition, the distance between R115 and E347 decreased, as did the distance between P102 and P107 under the PT116 + PT218 condition. Phosphorylation promoted the tilting of the protein pocket toward the active site, approaching a closed conformation, with overall changes ranging between 1 and 2 Å.

Comparatively, in the FECH-PPIX system, before phosphorylation, the distance between R115 and E343 increased compared with that in the FECH-Apo system, while the distances between R115 and E347 and between K118 and E351 decreased, leading the system to shift toward a closed conformation. After phosphorylation, the distance between R115 and E343 decreased, while the distances between R115 and E347 and between K118 and E351 tended to increase, indicating that phosphorylation drove the protein pocket away from the active site, leaning to an open conformation. Specifically, the distance between P102 and P107 increased in PT116, which was potentially related to the interaction between T116 and another α-helix [10].

In the analysis of the FECH-Heme system, a consistent trend was observed where the distance between R115 and E343 decreased while R115 and E347 increased, suggesting a tendency towards an open conformation due to phosphorylation. Particularly under the PT116 + PT218 condition, the distance between P102 and P107 significantly increased. Double-site phosphorylation in the FECH-Apo system had the smallest distance between the two lips among the four phosphorylated states, whereas in the FECH-PPIX system, this distance was further reduced. Conversely, in the FECH-Heme system, the distance actually increased compared to single-site phosphorylation. Overall, double-site phosphorylation markedly enhanced the protein structure’s ability to adjust, facilitating the catalytic reaction.

### 2.5. Binding Free Energy Analysis

To investigate the impact of FECH phosphorylation on substrate binding, the MM-PBSA method was used to calculate the binding free energy of PPIX and Heme to FECH in its unphosphorylated and phosphorylated states. Although the absolute binding free energy values may not be exact, this approach provides a reliable ranking system for assessing the ability of FECH to bind to its substrates in different phosphorylation states. The computed binding free energy ($∆G_{\mathrm{bind}}$) of the complex is composed of the van der Waals energy ($∆E_{\mathrm{vdW}}$), electrostatic energy ($∆E_{\mathrm{ele}}$), polar solvation energy ($∆G_{\mathrm{pol}/\mathrm{solv}}$), and nonpolar solvation energy ($∆G_{\mathrm{np}/\mathrm{solv}}$) (Table 3). These simulation results showed that the binding free energies of FECH with PPIX and Heme in UP were −55.39 kcal mol^−1^ and −66.49 kcal mol^−1^, respectively. For PT116, there was a slight increase in the binding affinity between FECH and the substrates (−57.49 kcal mol^−1^ and −66.60 kcal mol^−1^). In contrast, for PT218, a significant decrease in binding affinity was observed (−44.14 kcal mol^−1^ and −29.89 kcal mol^−1^). With respect to double-site phosphorylation (PT116 + PT218), the binding affinity between FECH and PPIX increased (−72.05 kcal mol^−1^), whereas that between FECH and Heme decreased (−50.81 kcal mol^−1^). In this state, phosphorylation enhanced PPIX binding and facilitated heme release.

Analysis of the energy component contributions revealed that the van der Waals interaction energy ($∆E_{\mathrm{vdW}}$), nonpolar solvation energy ($∆G_{\mathrm{np}/\mathrm{solv}}$), and electrostatic interaction energy ($∆E_{\mathrm{ele}}$) positively impacted ligand binding to FECH, whereas the polar solvation energy ($∆G_{\mathrm{pol}/\mathrm{solv}}$) had a negative effect on binding (Table 3). The contributions of PT218 to van der Waals, electrostatic, and nonpolar solvation energies were diminished compared with those of other phosphorylation states, resulting in weaker attractive interactions that disfavored PPIX binding. On the other hand, the contributions of PT116 and PT116 + PT218 were increased, thereby promoting PPIX binding. In both PT218 and PT116 + PT218 cases, the decrease in the electrostatic force contribution was more pronounced than that in the PT116 and UP cases. Furthermore, a greater reduction in non-solvation energy further promoted heme release.

### 2.6. Per-Residue Free Energy Decomposition Analysis

Energy decomposition techniques provide insightful information on complex substrate–receptor binding mechanisms [30]. In this study, MM-PBSA was applied to perform energy decomposition for individual residues, identifying key residues involved in substrate binding. Residues with an absolute interaction value with the substrate greater than 1 kcal mol^−1^ were considered essential for binding. The key residues were selected based on their interactions with the substrates.

**Figure 7:**
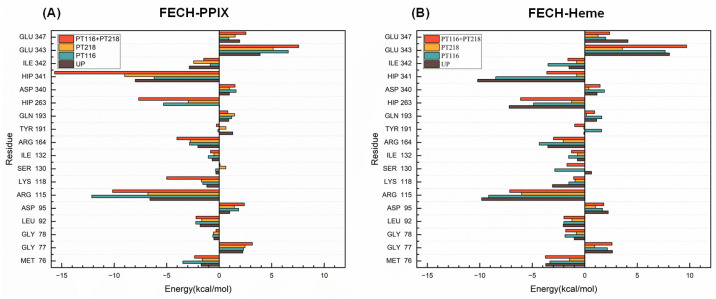
Binding free energy decomposition (kcal mol^−1^) of the important residues in the FECH-PPIX systems and FECH-Heme systems in four phosphorylation states. (**A**) The FECH-PPIX systems; (**B**) the FECH-Heme systems.

In the FECH-PPIX systems, residues including M76, R115, K118, R164, H263 and H341 exhibited increased attractive interactions, whereas E343 and E347 had inhibitory effects on binding. M76, R115, K118, R164, and H263 showed enhanced attractive interactions, but E343 had a higher inhibitory effect after phosphorylation (Figure 7A). The attractive contributions of M76 and R115 to binding were the largest in PT116, and those of R164, K118, H263 and H41 were the largest in PT116 + PT218. The inhibitory effects of E343 and E347 were the highest in PT116 + PT218.

In FECH-Heme systems, certain residues had attractive interactions and inhibitory effects like those in the FECH-PPIX systems (Figure 7B). After phosphorylation, the attractive interactions of M76, R115, K118, H263, and H341 were reduced, and the inhibitory effect of E343 was reduced, too. However, the R164 attractive interactions increased in PT116, and the inhibitory effect of E347 was increased in PT116 + PT218. Moreover, the reduced effect of these specific residues was most pronounced in PT218.

In short, phosphorylated FECH and its substrates (PPIX and Heme) exhibited a greater number of residues with favorable energy contribution changes and a relatively lower number of residues with unfavorable energy contribution changes. In summary, PT116 + PT218 emerged as the most optimal of all systems, displaying the highest affinity for PPIX binding to FECH protein, while showing lower affinity for heme binding compared to UP, which was conducive to enhancing the catalytic activity of FECH protein.

### 2.7. Protein–Substrate Interactions

Hydrogen bonding is a reliable indicator of bonding strength and provides insights into the degree of association between molecules [31]. It plays a crucial role in protein–substrate interactions by facilitating substrate binding to the active site of enzymes through specific amino acid residues [32]. Notably, S130, which can potentially be phosphorylated by kinases, has the potential to alter substrate interactions and affect FECH activity [33]. Several active site residues were found to interact with the substrate, specifically S130 and Y123, which form hydrogen bonds with propionate 6, and R115, which forms a salt bridge with propionate 7 [16].

**Figure 8:**
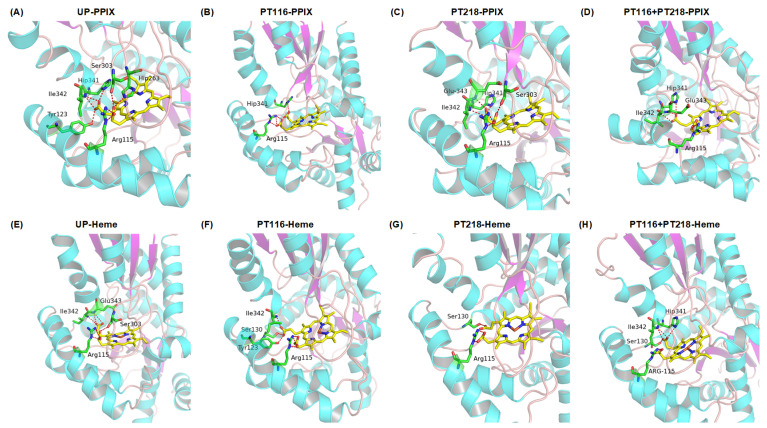
The interactions of the FECH residues bound with substrate (PPIX or Heme) in four phosphorylation states are shown in (**A**–**D**) the FECH-PPIX systems and (**E**–**H**) the FECH-Heme systems in MD simulations.

The intermolecular substrate–residue interactions at the active site of FECH are illustrated in Figure 8. In the UP state, R115, H263, and S303 formed hydrogen bonds with PPIX at position 6. Y130, H341, and I342 formed hydrogen bonds at position 7 (Figure 8A). After phosphorylation of T116, only the hydrogen bonds remained between R115 and position 6, H341 and position 7 (Figure 8B). The phosphorylation of T218 broke the hydrogen bond of H263 and Y123 with PPIX and added the bond of E343 with PPIX (Figure 8C). When both sites were phosphorylated, position 6 only connected with R115, and position 7 stayed the same, as in the case with PT218 (Figure 8D).

For the FECH-Heme systems, R115 and S303 formed hydrogen bonds with heme at position 6, and I342 and E343 formed hydrogen bonds at position 7 in the UP state (Figure 8E). Hydrogen bonds of H263, I342 and Y130 broke, and those of E343 formed, triggering movement of heme out of the active site. In the PT116 state, the bonds of E343 and S303 broke, and S130 and Y123 formed new hydrogen bonds. In the PT218 state, only the bonds between R115 and position 6 and between S130 and position 7 remained. In the PT116 + PT218 state, E343 and S303’s bonds broke, but S130 and H341’s bonds reformed. After phosphorylation, S130 formed hydrogen bonds with heme, indicating that S130 is involved in phosphorylation.

### 2.8. Principal Component Analysis

**Figure 9:**
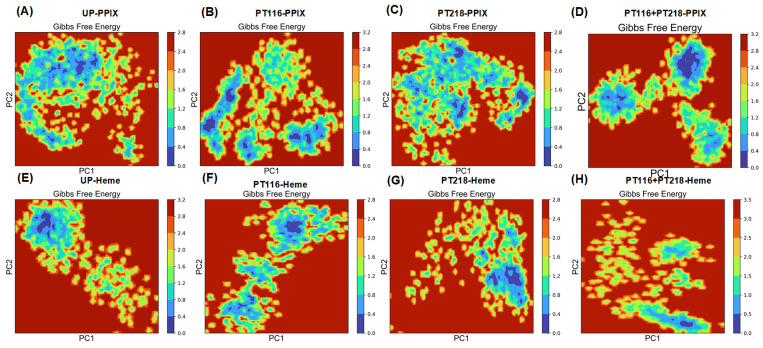
FELs generated by projecting the principal components PC1 and PC2 of systems in four phosphorylation states are shown in (**A**–**D**) the FECH-PPIX systems and (**E**–**H**) the FECH-Heme systems in MD simulations.

Principal component analysis (PCA) is a widely used computational method in MD simulations that provides valuable insights into the essential molecular motions and collective dynamics exhibited by biomolecules [34]. By projecting the system’s primary eigenvectors (PC1 and PC2), it is possible to calculate the Gibbs free energy landscape (FEL), which is a fundamental thermodynamic concept [35]. The accompanying images visually represent the Gibbs free energy landscape, with different colors indicating distinct energy states. This landscape is a powerful tool for investigating the directional fluctuations and conformational changes within FECH systems, considering the positions of all Cα atoms derived from the trajectories (Figure 9).

In the FECH-Apo systems (Figure S4), the motion correlation of PT218 exhibited a significantly higher magnitude, which is consistent with the results obtained from the RMSF analysis. The figure illustrates the FEL of the first two principal component complexes, showing the distinct lowest points for each complex. This observation suggested that the presence of PT218 induced significant conformational variations. In the FECH-PPIX systems, the FELs displayed scattered basins in the conformational space for PT116 and PT218. Notably, there was a more pronounced difference in the conformational space in PT116 and PT116 + PT218 (Figure 9B,D). For the FECH-Heme systems, local minima were distributed across approximately three to four regions within the energy landscape for both PT116 and PT116 + PT218 (Figure 9F,H), while UP and PT218 formed two to three metastable conformations throughout the trajectory population (Figure 9E,G). In conclusion, the phosphorylation of residues caused a redistribution of the conformational space of the substrates binding FECH proteins.

### 2.9. Dynamic Cross-Correlation Matrix

**Figure 10:**
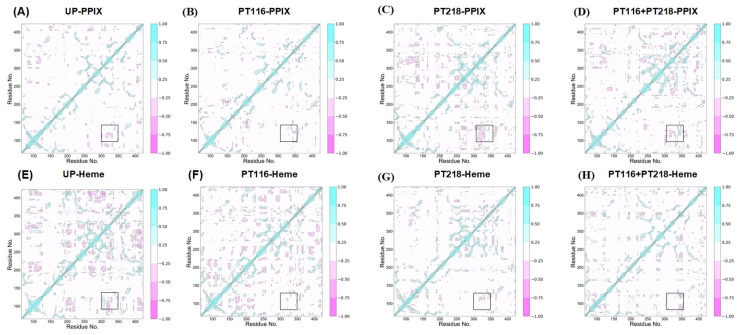
Dynamic cross-correlation maps of the fluctuations in the coordinates of the Cα atoms around the mean positions of the systems in the four phosphorylation states are shown for the (**A**–**D**) FECH-PPIX systems and (**E**–**H**) FECH-Heme systems. The extent of correlated and anticorrelated motions is color-coded (1 = highly correlated; 0 = not correlated; −1 = inversely correlated).

Dynamic cross-correlation matrix (DCCM) analysis was employed to analyze the position of the Cα atom during simulated conformational changes, providing insights into correlated motion [36]. The colors ranging from white to cyan represent highly correlated movements, and the transition from white to pink indicates noncorrelated movements (Figure 10). The results showed that all systems consistently exhibited correlated motion. In the FECH-Apo systems, phosphorylation increased the correlation between residues, particularly showing a strong negative correlation between the π-helix and upper lip region in PT218. This finding aligns with the RMSF results, which indicated greater fluctuations in the upper lip region for PT218. Regions with increased amino acid fluctuations demonstrated stronger correlations with movement (Figure S5). In the FECH-PPIX systems, phosphorylation led to varying degrees of reduction in the correlation between the π-helix and upper lip region. The most significant decrease was observed in PT116 (Figure 10B), whereas PT218 did not experience a substantial decrease (Figure 10C), and PT116 + PT218 experienced a moderate decrease (Figure 10D). In the FECH-Heme systems, phosphorylation resulted in an overall reduction in residue correlation. Notably, PT218 displayed a more pronounced decrease in correlation and weaker interactions, indicating a favorable environment for heme release (Figure 10E–H). In short, the phosphorylation of residues significantly altered the dynamic characteristics of local residues on substrates (PPIX and Heme) and FECH proteins and affected the inter-residue correlation and anti-correlation movements.

## 3. Materials and Methods

### 3.1. Cloning, Expression, and Purification of Phosphorylated Human FECH

The human *FECH* and *PKA* genes were cloned into the pETDuet plasmid (a gift from the Michael Terns Laboratory, The University of Georgia) in sequential order. *FECH* and *T116A FECH*, which was PCR amplified from a pBTac-FECH vector [37], were first cloned into EcoRI/HindIII sites of the pETDuet plasmid. PKA, amplified from a commercially available vector (DNASU Plasmid Repository, Clone ID: HsCD00343196), was cloned into NdeI/FseI sites of the resulting pETDuet-FECH/PKA vector. The inserts were confirmed by Sanger sequencing. The pETDuet-FECH/PKA vector yielded a 6x His-tagged FECH protein and a non-His-tagged PKA, as designed.

BL21(DE3) *E. coli* (New England Biolabs) or BL21(DE3) Δ*ycdX E. coli* [38] were transformed with the pETDuet-FECH/PKA vector. The transformed cells were cultured overnight in 100 mL of Terrific Broth (TB) media supplemented with ampicillin (50 µg/mL final concentration). An overnight culture was used to inoculate 1 L of TB media, supplemented with 0.6 g of glucose, 3 g of lactose, and ampicillin (50 µg/mL final concentration). The 1 L culture was initially grown at 30 °C and 220 rpm for 1 h, after which the temperature was reduced to 18 °C, and the culture was allowed to grow for an additional 47 h. The cells were harvested and stored at −80 °C. For protein purification, cell pellets were resuspended and lysed, and FECH was purified as described here [39]. The eluted protein was dialyzed in a 6 M urea 50 mM Tris-Mops buffer and concentrated to ~3 mg/mL.

### 3.2. Mass Spectrometry Analysis of Purified FECH Protein

Purified FECH was buffer exchanged in 40 mM ammonium bicarbonate (Sigma, St. Louis, Missouri. USA) via 10 kDa molecular weight cut-off (MWCO) filter, reduced by incubating with 10 mM of dithiothreitol (Sigma) at 56 °C and alkylated by 27.5 mM of iodoacetamide (Sigma) at room temperature in dark. The reduced and alkylated proteins were divided into two aliquots: one aliquot was digested by trypsin (Promega, Madison, WI, USA) at 37 °C, and the other aliquot was digested by chymotrypsin (Promega) at 25 °C. The resulting peptides from the respective enzymatic digestions were separated on an Acclaim PepMap RSLC C18 column (75 µm × 15 cm) and eluted into the nano-electrospray ion source of an Orbitrap Fusion™ Tribrid™ mass spectrometer (Thermo Fisher Scientific, Waltham, MA, USA) at a flow rate of 200 nL/min. The elution gradient consisted of 1–40% acetonitrile in 0.1% formic acid over 220 min, followed by 10 min of 80% acetonitrile in 0.1% formic acid. The spray voltage was set to 2.2 kV, and the temperature of the heated capillary was set to 275 °C. Full MS scans were acquired from *m*/*z* 200 to 2000 at 60 k resolution, and MS/MS scans following collision-induced dissociation (CID) at 38% collision energy or electron transfer dissociation (ETD) were collected in the ion trap. The raw spectra were analyzed using Proteome Discoverer (Thermo Fisher Scientific) and Byonic (Protein Metrics, Cupertino, CA, USA) [40] with the mass tolerance set as 20 ppm for precursors and 0.5 Da for fragments. The search output was filtered to reach a 1% false discovery rate and then validated manually for any phosphorylation sites assigned by the program. The occupancy of each phosphorylation site was calculated using spectral counts assigned to the phosphorylated peptides and their unmodified counterparts.

### 3.3. System Modeling

MD simulations are valuable scientific tools for studying protein dynamics and investigating the impact of PTMs on protein behavior. The initial structure utilized in this study was derived from the crystal structure of human FECH, which contained the R115L variant (PDB 2HRC) [16]. Additionally, the initial structure of human FECH in complex with PPIX was constructed from the E343K variant (PDB: 2QD1) [11], and the complex with heme was built from the F110A variant (PDB: 2QD2) [11]. All the mutated residues present in the crystal structures were changed back to the wild-type sequence.

The protonation state of the histidine residues (HID or HIE) was determined by calculating protonation equilibria using the H++ server [41]. The parameter files and the electrostatic potential (ESP) charge of the porphyrin and heme were generated using Turbomole 7.6 software [42] at the level of BP86/6-31 G* for all atoms (Fe was described by the DZpdf basis set) [43].

MD simulations were conducted using the pmemd.cuda module of the Amber 20 software package [44]. The systems were characterized using the ff19SB force field [45] and the OPC explicit solvent model [46]. The phosphorylated threonine was characterized using the phosaa19 force field [47]. The coordination of the [2Fe-2S] cluster involved Fe^2+^ binding with two central sulfur atoms, as well as two additional sulfur atoms from CYS residues. Specifically, FE1 coordinated with 196SG and 403SG, while FE2 coordinated with 406SG and 411SG. Cl^−^ and Na^+^ ions were added as needed to maintain overall system neutrality. The SHAKE algorithm [48] was used to constrain hydrogen atom bonds. The system pressure was maintained at 1 atm, and the temperature was maintained at 300 K using a Langevin thermostat [49]. Long-range electrostatic interactions were calculated using the particle-mesh Ewald method [50], with a nonbonding interaction cutoff distance of 9 Å.

Three steps of minimization were performed to relax the solvent molecules and protein–ligand complexes. First, only the water molecules in the systems were minimized, followed by the minimization of the side chains of residues, and finally, all atoms were minimized. Subsequently, each system was gradually heated from 0 K to 300 K under the NVT ensemble. The systems were then simulated for 5 ns under the NVT ensemble, with positional restraints set at 5 kcal mol^−1^ Å^−2^. Next, the systems were equilibrated under the NPT ensemble at 1 atm for 500 ps. Finally, production runs were conducted for 1 μs under the NPT ensemble, using the Berendsen barostat [51], without any restraints. A time-step of 2 fs was employed, and snapshots were saved every 1 ns, resulting in a total of 1000 snapshots.

### 3.4. Binding Free Energy Calculations

The binding free energies between the substrates (PPIX and Heme) and the protein were calculated using the Poisson–Boltzmann surface area (MM-PBSA) method [52,53,54] in Amber20. The binding free energies $∆G_{\mathrm{bind}}\;$of a specific substrate were averaged over the last 100 ns of MD trajectories (consisting of 100 frames) using the following three equations:

(1) $$∆G_{\mathrm{bind}}=∆E_{\mathrm{MM}}+∆G_{\mathrm{solv}}\;-T∆S$$

(2) $$∆E_{\mathrm{MM}}=∆E_{\mathrm{vdW}}+∆E_{\mathrm{ele}}+∆E_{\mathrm{bond}}$$

(3) $$∆G_{\mathrm{solv}}=∆G_{\mathrm{pol}/\mathrm{solv}}+∆G_{\mathrm{np}/\mathrm{solv}}$$

The energy variation ($∆E_{\mathrm{MM}}$) included the combined effects of both bonded interactions ($∆E_{\mathrm{bond}}$) and nonbonded interactions ($∆E_{\mathrm{vdW}}$ and $∆E_{\mathrm{ele}}$). In a single-trajectory setup, the influence of bonded interactions ($∆E_{\mathrm{bond}}$) was consistently neglected because its accuracy and simplicity were comparable to those of multi-trajectory approaches. The determination of solvation free energy (∆Gsol) involved the comprehensive assessment of both polar solvation energy ($∆G_{\mathrm{pol}/\mathrm{solv}}$) and nonpolar solvation energy ($∆G_{\mathrm{np}/\mathrm{solv}}$). These energies were calculated using the Poisson–Boltzmann equation and the solvent-accessible surface area model [55]. The default parameter configurations for these computations were consistent with those in the established literature.

### 3.5. Molecular Dynamics Trajectory Analysis

The resulting trajectories were analyzed using the CPPTRAJ [56] module of Amber 20. Principal component analysis (PCA) is a technique that reduces the dimensionality of MD data while preserving structural information [57,58]. The Bio3d package [59] in R Studio 4.2.3 was used to gain insights into protein dynamics extracted from MD trajectories by performing PCA. Dynamic cross-correlation matrix (DCCM) analysis was applied to investigate changes in Cα atoms, their fluctuations, and their movements. The details of PCA and DCCM methods can be seen in Supporting Information. The matrices were measured and plotted using Python 3.7. Additionally, PyMOL 3.0 [60] and VMD 1.9.4 [61] software were used for trajectory visualization and observing structural details.

## 4. Conclusions

In this study, we investigated the dynamic properties of FECH under four distinct post-translational phosphorylation states: unmodified UP, single-site PT116 and PT218, and double-site modification PT116 + PT218, both in the absence and presence of substrates (PPIX or Heme). Our approach commenced with stabilizing 12 complex systems through molecular dynamics simulations, which included FECH in varying phosphorylation states interacting with and without substrates. The binding free energies between FECH and the substrates were subsequently analyzed using the MM-PBSA method. Our findings revealed that the PT116 + PT218 state exhibited the lowest binding free energy with PPIX, indicating the strongest binding affinity. This state also exhibited a higher binding free energy when interacting with Heme compared to UP, facilitating Heme release. These results highlight the PT116 + PT218 state as particularly advantageous for enhancing FECH protein catalytic activity.

To further elucidate the interactions between FECH and the substrates, we performed energy decomposition and interaction analysis at the residue level. These analyses highlighted that variations in electrostatic energies were primarily responsible for differences in binding affinity across all systems. Additionally, by integrating multiple analytical methods, we clarified how phosphorylation of residues leads to structural changes within FECH. Specifically, phosphorylation altered the conformational space of the substrate-binding sites and significantly enhanced the collective movements of active pocket residues, as well as the correlated and anti-correlated motions within the complex.

In conclusion, this research illuminates the mechanisms of substrate–protein interactions under different phosphorylated states and demonstrates why double-point phosphorylation is beneficial for activity enhancement. The insights gained from this study may provide valuable theoretical guidance for treating conditions associated with disrupted heme metabolism, such as various porphyrias and iron-related disorders.

## Figures and Tables

**Table 1:** Mean Cα RMSDs during the 1 μs simulations of the FECH-Apo systems, the FECH-PPIX systems and the FECH-heme systems in four phosphorylation states.

Systems	Phosphorylated Sites	Mean RMSD(SD) (Å)
FECH-Apo	UP	2.19 (0.19)
	PT116	1.98 (0.15)
	PT218	2.07 (0.26)
	PT116 + PT218	2.19 (0.24)
FECH-PPIX	UP	1.79 (0.17)
	PT116	2.06 (0.18)
	PT218	2.17 (0.20)
	PT116 + PT218	2.30 (0.17)
FECH-Heme	UP	2.39 (0.25)
	PT116	2.22 (0.18)
	PT218	2.10 (0.17)
	PT116 + PT218	2.11 (0.19)

**Table 2:** Average interaction distances between selected residue pairs during the last 100 ns MD simulations in the FECH-Apo systems, the FECH-PPIX systems and the FECH-Heme systems for the four phosphorylation states.

System	Phosphorylation Site	Residue Pairs Mean Distance (SD) (Å)			
		P102–P107	R115–E343	R115–E347	K118–E351
FECH-Apo	UP	6.56 (1.49)	9.78 (2.28)	8.10 (2.11)	7.29 (1.88)
	PT116	7.32 (1.61)	10.17 (2.24)	7.85 (2.28)	6.93 (2.53)
	PT218	7.34 (3.90)	11.55 (2.40)	10.03 (2.51)	7.10 (2.51)
	PT116 + PT218	5.00 (2.36)	10.59 (1.89)	9.10 (1.81)	5.65 (2.16)
FECH-PPIX	UP	6.05 (1.38)	12.7 (0.73)	7.13 (0.83)	5.56 (1.48)
	PT116	6.70 (1.42)	9.82 (3.39)	8.16 (1.20)	6.76 (1.69)
	PT218	5.64 (1.42)	10.13 (2.27)	7.82 (0.84)	6.16 (1.47)
	PT116 + PT218	4.68 (1.21)	7.23 (1.03)	10.09 (1.12)	7.98 (1.76)
FECH-Heme	UP	6.84 (1.45)	11.60 (1.12)	7.10 (1.02)	5.91 (2.25)
	PT116	6.43 (1.38)	8.31 (0.58)	8.41 (0.81)	6.88 (2.16)
	PT218	6.08 (1.43)	8.34 (2.50)	7.68 (1.05)	5.77 (1.48)
	PT116 + PT218	8.17 (1.53)	9.82 (2.95)	8.23 (0.90)	7.59 (2.59)

**Table 3:** Energetic components of the binding free energy of FECH bound with PPIX or Heme over the last 100 ns of MD trajectories in four phosphorylation states.

Component	$∆E_{\mathrm{vdW}}$	$∆E_{\mathrm{ele}}$	$∆G_{\mathrm{pol}/\mathrm{solv}}$	$∆G_{\mathrm{np}/\mathrm{solv}}$	$∆G_{\mathrm{gas}}$	$∆G_{\mathrm{solv}}$	$∆G_{\mathrm{bind}}$
UP-PPIX	−65.86 (1.98)	−396.00 (10.41)	412.53 (10.88)	−6.07 (0.18)	−461.86 (12.34)	406.47 (10.71)	−55.39 (1.76)
PT116-PPIX	−71.70 (0.29)	−317.58 (1.96)	338.30 (1.69)	−6.51 (0.01)	−389.28 (1.97)	331.79 (1.69)	−57.49 (0.71)
PT218-PPIX	−57.22 (3.06)	−306.88 (14.65)	325.08 (15.62)	−5.12 (17.67)	−364.10 (15.35)	319.96 (2.45)	−44.14 (1.45)
PT116 + PT218-PPIX	−72.58 (0.33)	−312.86 (1.74)	319.78 (1.55)	−6.39 (0.01)	−385.44 (1.76)	313.39 (1.55)	−72.05 (0.76)
UP-Heme	−64.22 (2.16)	−374.72 (10.80)	378.33 (10.90)	−5.88 (0.20)	−438.94 (12.91)	372.45 (10.71)	−66.49 (2.31)
PT116-Heme	−72.15 (1.51)	−292.46 (5.50)	304.26 (5.74)	−6.25 (0.13)	−364.61 (6.94)	298.01 (5.62)	−66.60 (1.52)
PT218-Heme	−46.09 (3.62)	−220.75 (14.66)	241.00 (16.29)	−4.05 (0.32)	−266.84 (18.26)	236.95 (15.97)	−29.89 (2.37)
PT116 + PT218-Heme	−69.21 (1.93)	−250.66 (6.54)	275.07 (7.15)	−6.01 (0.16)	−319.87 (8.38)	269.07 (6.99)	−50.81 (1.65)

## Data Availability

The original contributions presented in the study are included in the article/Supplementary Material, further inquiries can be directed to the corresponding author/s.

## Supplementary Materials

The following supporting information and Table S1 can be downloaded at: https://www.mdpi.com/article/10.3390/ijms25126360/s1. Reference [62] is cited in the supplementary materials.

## References

[1] Burris T.P. (2008). Nuclear Hormone Receptors for Heme: REV-ERBalpha and REV-ERBbeta Are Ligand-Regulated Components of the Mammalian Clock. Mol. Endocrinol..

[2] Faller M., Matsunaga M., Yin S., Loo J.A., Guo F. (2007). Heme Is Involved in microRNA Processing. Nat. Struct. Mol. Biol..

[3] Hu R.-G., Wang H., Xia Z., Varshavsky A. (2008). The N-End Rule Pathway Is a Sensor of Heme. Proc. Natl. Acad. Sci. USA.

[4] Shen J., Sheng X., Chang Z., Wu Q., Wang S., Xuan Z., Li D., Wu Y., Shang Y., Kong X. (2014). Iron Metabolism Regulates P53 Signaling through Direct Heme-P53 Interaction and Modulation of P53 Localization, Stability, and Function. Cell Rep..

[5] Burton M.J., Kapetanaki S.M., Chernova T., Jamieson A.G., Dorlet P., Santolini J., Moody P.C.E., Mitcheson J.S., Davies N.W., Schmid R. (2016). A Heme-Binding Domain Controls Regulation of ATP-Dependent Potassium Channels. Proc. Natl. Acad. Sci. USA.

[6] Sahoo N., Goradia N., Ohlenschläger O., Schönherr R., Friedrich M., Plass W., Kappl R., Hoshi T., Heinemann S.H. (2013). Heme Impairs the Ball-and-Chain Inactivation of Potassium Channels. Proc. Natl. Acad. Sci. USA.

[7] Al-Karadaghi S., Franco R., Hansson M., Shelnutt J.A., Isaya G., Ferreira G.C. (2006). Chelatases: Distort to Select?. Trends Biochem. Sci..

[8] Medlock A.E., Dailey H.A. (2022). New Avenues of Heme Synthesis Regulation. Int. J. Mol. Sci..

[9] Dailey H.A., Medlock A.E. (2022). A Primer on Heme Biosynthesis. Biol. Chem..

[10] Obi C.D., Bhuiyan T., Dailey H.A., Medlock A.E. (2022). Ferrochelatase: Mapping the Intersection of Iron and Porphyrin Metabolism in the Mitochondria. Front. Cell Dev. Biol..

[11] Medlock A.E., Dailey T.A., Ross T.A., Dailey H.A., Lanzilotta W.N. (2007). A π-Helix Switch Selective for Porphyrin Deprotonation and Product Release in Human Ferrochelatase. J. Mol. Biol..

[12] Balwani M., Doheny D., Bishop D.F., Nazarenko I., Yasuda M., Dailey H.A., Anderson K.E., Bissell D.M., Bloomer J., Bonkovsky H.L. (2013). Loss-of-Function Ferrochelatase and Gain-of-Function Erythroid-Specific 5-Aminolevulinate Synthase Mutations Causing Erythropoietic Protoporphyria and X-Linked Protoporphyria in North American Patients Reveal Novel Mutations and a High Prevalence of X-Linked Protoporphyria. Mol. Med..

[13] Wensink D., Wagenmakers M.A.E.M., Qi H., Wilson J.H.P., Langendonk J.G. (2022). Objective Light Exposure Measurements and Circadian Rhythm in Patients with Erythropoietic Protoporphyria: A Case-Control Study. Mol. Genet. Metab..

[14] Dailey H.A., Meissner P.N. (2013). Erythroid Heme Biosynthesis and Its Disorders. CSH. Perspect. Med..

[15] Chen F.-P., Risheg H., Liu Y., Bloomer J. (2002). Ferrochelatase Gene Mutations in Erythropoietic Protoporphyria: Focus on Liver Disease. Cell. Mol. Biol..

[16] Medlock A., Swartz L., Dailey T.A., Dailey H.A., Lanzilotta W.N. (2007). Substrate Interactions with Human Ferrochelatase. Proc. Natl. Acad. Sci. USA.

[17] Medlock A.E., Carter M., Dailey T.A., Dailey H.A., Lanzilotta W.N. (2009). Product Release Rather than Chelation Determines Metal Specificity for Ferrochelatase. J. Mol. Biol..

[18] Deribe Y.L., Pawson T., Dikic I. (2010). Post-Translational Modifications in Signal Integration. Nat. Struct. Mol. Biol..

[19] Theillet F.-X., Smet-Nocca C., Liokatis S., Thongwichian R., Kosten J., Yoon M.-K., Kriwacki R.W., Landrieu I., Lippens G., Selenko P. (2012). Cell Signaling, Post-Translational Protein Modifications and NMR Spectroscopy. J. Biomol. NMR.

[20] Chung J., Wittig J.G., Ghamari A., Maeda M., Dailey T.A., Bergonia H., Kafina M.D., Coughlin E.E., Minogue C.E., Hebert A.S. (2017). Erythropoietin Signaling Regulates Heme Biosynthesis. eLife.

[21] Chung J., Chen C., Paw B.H. (2012). Heme Metabolism and Erythropoiesis. Curr. Opin. Hematol..

[22] Tanaka T., Nangaku M. (2012). Recent Advances and Clinical Application of Erythropoietin and Erythropoiesis-Stimulating Agents. Exp. Cell. Res..

[23] Feng G., Zhang X., Li Y., Wang R. (2022). Analysis of the Binding Sites on BAX and the Mechanism of BAX Activators through Extensive Molecular Dynamics Simulations. J. Chem. Inf. Model..

[24] Maloney R.C., Zhang M., Liu Y., Jang H., Nussinov R. (2022). The Mechanism of Activation of MEK1 by B-Raf and KSR1. Cell. Mol. Life Sci..

[25] Duff N., Peters B. (2011). Polymorph Specific RMSD Local Order Parameters for Molecular Crystals and Nuclei: α-, β-, and γ-Glycine. J. Chem. Phys..

[26] Kokkinidis M., Glykos N.M., Fadouloglou V.E. (2012). Protein Flexibility and Enzymatic Catalysis. Advances in Protein Chemistry and Structural Biology.

[27] Kuzmanic A., Zagrovic B. (2010). Determination of Ensemble-Average Pairwise Root Mean-Square Deviation from Experimental B-Factors. Biophys. J..

[28] Wang S., Peng J., Ma J., Xu J. (2016). Protein Secondary Structure Prediction Using Deep Convolutional Neural Fields. Sci. Rep..

[29] Wang Y., Wu J., Ju J., Shen Y. (2013). Investigation by MD Simulation of the Key Residues Related to Substrate-Binding and Heme-Release in Human Ferrochelatase. J. Mol. Model..

[30] Zhou Y., Jiang Y., Chen S. (2022). RNA –Ligand Molecular Docking: Advances and Challenges. WIREs Comput. Mol. Sci..

[31] Hubbard R.E., Kamran Haider M. (2010). Hydrogen Bonds in Proteins: Role and Strength. eLS.

[32] Patil R., Das S., Stanley A., Yadav L., Sudhakar A., Varma A.K. (2010). Optimized Hydrophobic Interactions and Hydrogen Bonding at the Target-Ligand Interface Leads the Pathways of Drug-Designing. PLoS ONE.

[33] Sakaino M., Ishigaki M., Ohgari Y., Kitajima S., Masaki R., Yamamoto A., Taketani S. (2009). Dual Mitochondrial Localization and Different Roles of the Reversible Reaction of Mammalian Ferrochelatase. FEBS J..

[34] David C.C., Jacobs D.J., Livesay D.R. (2014). Principal Component Analysis: A Method for Determining the Essential Dynamics of Proteins. Protein Dynamics.

[35] Granato D., Santos J.S., Escher G.B., Ferreira B.L., Maggio R.M. (2018). Use of Principal Component Analysis (PCA) and Hierarchical Cluster Analysis (HCA) for Multivariate Association between Bioactive Compounds and Functional Properties in Foods: A Critical Perspective. Trends Food Sci. Technol..

[36] Yu H., Dalby P.A. (2020). A. A Beginner’s Guide to Molecular Dynamics Simulations and the Identification of Cross-Correlation Networks for Enzyme Engineering. Methods in Enzymology.

[37] Dailey H.A., Sellers V.M., Dailey T.A. (1994). Mammalian Ferrochelatase. Expression and Characterization of Normal and Two Human Protoporphyric Ferrochelatases. J. Biol. Chem..

[38] Zhang M.S., Brunner S.F., Huguenin-Dezot N., Liang A.D., Schmied W.H., Rogerson D.T., Chin J.W. (2017). Biosynthesis and Genetic Encoding of Phosphothreonine through Parallel Selection and Deep Sequencing. Nat. Methods.

[39] Burden A.E., Wu C., Dailey T.A., Busch J.L., Dhawan I.K., Rose J.P., Wang B., Dailey H.A. (1999). Human Ferrochelatase: Crystallization, Characterization of the [2Fe-2S] Cluster and Determination That the Enzyme Is a Homodimer. Biochim. Biophys. Acta.

[40] Bern M., Kil Y.J., Becker C. (2012). Byonic: Advanced Peptide and Protein Identification Software. Curr. Protoc. Bioinform..

[41] Anandakrishnan R., Aguilar B., Onufriev A.V. (2012). H++ 3.0: Automating pK Prediction and the Preparation of Biomolecular Structures for Atomistic Molecular Modeling and Simulations. Nucleic Acids Res..

[42] Ahlrichs R., Bär M., Häser M., Horn H., Kölmel C. (1989). Electronic Structure Calculations on Workstation Computers: The Program System Turbomole. Chem. Phys. Lett..

[43] Sigfridsson E., Ryde U. (2003). The Importance of Porphyrin Distortions for the Ferrochelatase Reaction. J. Biol. Inorg. Chem..

[44] Lee T.-S., Allen B.K., Giese T.J., Guo Z., Li P., Lin C., McGee T.D., Pearlman D.A., Radak B.K., Tao Y. (2020). Alchemical Binding Free Energy Calculations in AMBER20: Advances and Best Practices for Drug Discovery. J. Chem. Inf. Model..

[45] Tian C., Kasavajhala K., Belfon K.A.A., Raguette L., Huang H., Migues A.N., Bickel J., Wang Y., Pincay J., Wu Q. (2020). ff19SB: Amino-Acid-Specific Protein Backbone Parameters Trained against Quantum Mechanics Energy Surfaces in Solution. J. Chem. Theory Comput..

[46] Xiong Y., Shabane P.S., Onufriev A.V. (2020). Melting Points of OPC and OPC3 Water Models. ACS Omega.

[47] Homeyer N., Horn A.H.C., Lanig H., Sticht H. (2006). AMBER Force-Field Parameters for Phosphorylated Amino Acids in Different Protonation States: Phosphoserine, Phosphothreonine, Phosphotyrosine, and Phosphohistidine. J. Mol. Model..

[48] Ryckaert J.-P., Ciccotti G., Berendsen H.J.C. (1977). Numerical Integration of the Cartesian Equations of Motion of a System with Constraints: Molecular Dynamics of n-Alkanes. J. Comput. Phys..

[49] Pastor R.W., Brooks B.R., Szabo A. (1988). An Analysis of the Accuracy of Langevin and Molecular Dynamics Algorithms. Mol. Phys..

[50] Darden T., York D., Pedersen L. (1993). Particle Mesh Ewald: An *N* log(*N*) Method for Ewald Sums in Large Systems. J. Chem. Phys. S.

[51] Berendsen H.J.C., Postma J.P.M., Van Gunsteren W.F., DiNola A., Haak J.R. (1984). Molecular Dynamics with Coupling to an External Bath. J. Chem. Phys..

[52] Kollman P.A., Massova I., Reyes C., Kuhn B., Huo S., Chong L., Lee M., Lee T., Duan Y., Wang W. (2000). Calculating Structures and Free Energies of Complex Molecules: Combining Molecular Mechanics and Continuum Models. Acc. Chem. Res..

[53] Srivastava H.K., Sastry G.N. (2012). Molecular Dynamics Investigation on a Series of HIV Protease Inhibitors: Assessing the Performance of MM-PBSA and MM-GBSA Approaches. J. Chem. Inf. Model..

[54] Kar P., Seel M., Hansmann U.H.E., Höfinger S. (2007). Dispersion Terms and Analysis of Size- and Charge Dependence in an Enhanced Poisson−Boltzmann Approach. J. Phys. Chem. B.

[55] Sitkoff D., Sharp K.A., Honig B. (1994). Accurate Calculation of Hydration Free Energies Using Macroscopic Solvent Models. J. Phys. Chem..

[56] Roe D.R., Cheatham T.E. (2013). PTRAJ and CPPTRAJ: Software for Processing and Analysis of Molecular Dynamics Trajectory Data. J. Chem. Theory Comput..

[57] Ichiye T., Karplus M. (1991). Collective Motions in Proteins: A Covariance Analysis of Atomic Fluctuations in Molecular Dynamics and Normal Mode Simulations. Proteins.

[58] Amadei A., Linssen A.B.M., De Groot B.L., Van Aalten D.M.F., Berendsen H.J.C. (1996). An Efficient Method for Sampling the Essential Subspace of Proteins. J. Biomol. Struct. Dyn..

[59] Grant B.J., Rodrigues A.P.C., ElSawy K.M., McCammon J.A., Caves L.S.D. (2006). Bio3d: An R Package for the Comparative Analysis of Protein Structures. Bioinformatics.

[60] Yuan S., Chan H.C.S., Hu Z. (2017). Using PyMOL as a Platform for Computational Drug Design. WIREs Comput. Mol. Sci..

[61] Humphrey W., Dalke A., Schulten K. (1996). VMD: Visual Molecular Dynamics. J. Mol. Graph..

[62] Maisuradze G.G., Liwo A., Scheraga H.A. (2010). Relation between Free Energy Landscapes of Proteins and Dynamics. J. Chem. Theory Comput..

