# Supplementary material for: Impact of Phosphorylation at Various Sites on the Active Pocket of Human Ferrochelatase: Insights from Molecular Dynamics Simulations
Source: Int J Mol Sci. 2024 Jun 8;25(12):6360. doi: 10.3390/ijms25126360 (PMC11203519; doi:10.3390/ijms25126360)
Supplement: Supplementary file 1 [file ijms-25-06360-s001.zip › Supporting information.pdf]

Supporting Information for

Impact of Phosphorylation at Various Sites on the Active Pocket of Human Ferrochelatase: Insights from Molecular Dynamics Simulations

Mingshan Guo [1], Yuhong Lin [1], Chibuike David Obi [2,†], Peng Zhao [3], Harry A. Dailey [2], Amy E. Medlock [2,4] and Yong Shen [1,*]

[1] School of Chemistry, IGCME, Sun Yat-sen University, Guangzhou 510006, China
[2] Department of Biochemistry and Molecular Biology, University of Georgia, Athens, GA 30602, USA; chibuike.obi@cchmc.org (C.D.O.); hdailey@uga.edu (H.A.D.); medlock@uga.edu (A.E.M.)
[3] Complex Carbohydrate Research Center, University of Georgia, Athens, GA 30602, USA; pengzhao@ccrc.uga.edu
[4] Augusta University/University of Georgia Medical Partnership, Athens, GA 30602, USA
* Correspondence: cessy@mail.sysu.edu.cn
† Current address: Division of Hematology, Cincinnati Children's Hospital Medical Center, Cincinnati, OH 45229, USA.

Methods

Principal Component Analysis

An appropriate conformational sampling procedure allows the derivation of the energy landscape for the conformational changes in protein-substrate complex. Conformations generated through MD simulations were employed for energy analysis. Free energy landscape (FEL) is a powerful technique for uncovering protein conformational changes linked to various energy states[1]. By examining the FEL minima, which indicate the system's stable states, and the FEL barriers, which correspond to transient states, FEL offers insights into the recognition, aggregation, and folding of biomolecules as well as the dynamic processes in biological systems. This approach helps to elucidate the steady and transitional behaviors of biomolecular structures. For a two-dimensional representation of the energy landscape, project the energy landscapes onto the first (PC1) and second (PC2) principal components, utilizing the highest eigenvalues derived from PCA analysis for FECH-substrates complexes at 300 K. Define the free energy landscapes as:

$$G_{(\mathrm{PC1,PC2})} = -k_{\mathrm{B}}T\ln\ P_{(\mathrm{PC1,PC2})},$$

where G represents the Gibbs free energy, k_{B} represents the Boltzmann constant, T is the absolute temperature, and $P_{(\mathrm{PC1,PC2})}$ is the normalized joint probability distribution.

Dynamic cross-correlation matrix analysis

Dynamic cross-correlation matrix (DCCM) analysis is crucial for comprehending protein internal dynamics[2]. It offers insights into the time correlation among protein residues i and j and is commonly employed to identify dynamic correlation variations across various systems. In MD simulations, DCCM reveals correlations in the movements of protein residue pairs. The DCCM can be expressed in terms of the cross-correlation coefficient C_{ij} and is defined by the following formula:

$$\mathrm{C}_{\mathrm{j}}^{\mathrm{i}} = \frac{<\Delta \mathrm{r}_{\mathrm{i}} \cdot \Delta \mathrm{j}_{\mathrm{i}}>}{\sqrt{<\Delta \mathrm{r}_{\mathrm{i}}>^{2} \cdot <\Delta \mathrm{j}_{\mathrm{i}}>^{2}}}$$

Δr_i and Δj_i represent the displacements of residue i and residue j. A positive C_{ij}represents positive correlation motion, while a negative value represents negative correlation between residue i and residue j.

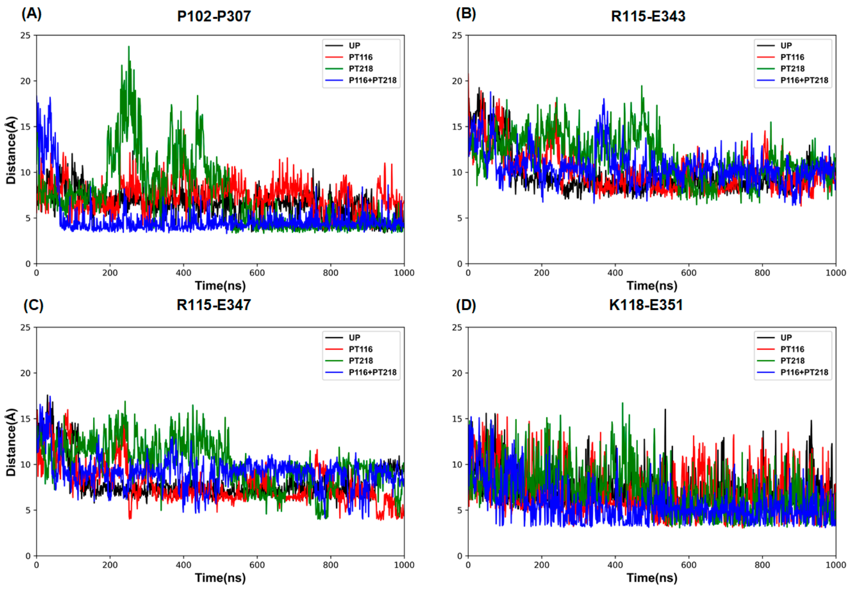

Figure S1. Time evolutions of the interaction distances between selected residue pairs during the 1000 ns MD simulations of FECH-Apo systems. (A) the distance between P102 and P107, (B) the interaction distance between R115 and E343 (C) the interaction distance between R115 and E347, (D) the interaction distance between K118 and E351 .

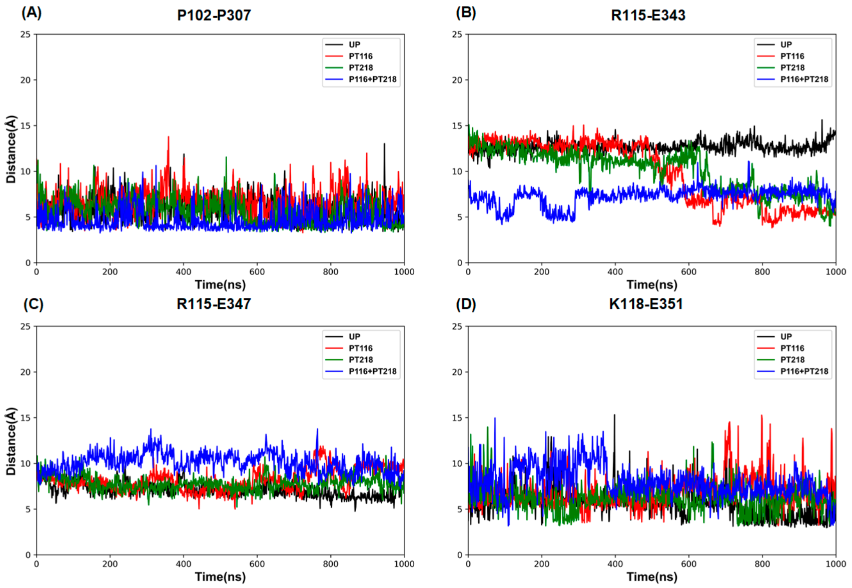

Figure S2. Time evolutions of the interaction distances between selected residue pairs during the 1000 ns MD simulations of FECH-PPIX systems. (A) the distance between P102 and P107, (B) the interaction distance between R115 and E343 (C) the interaction distance between R115 and E347, (D) the interaction distance between K118 and E351.

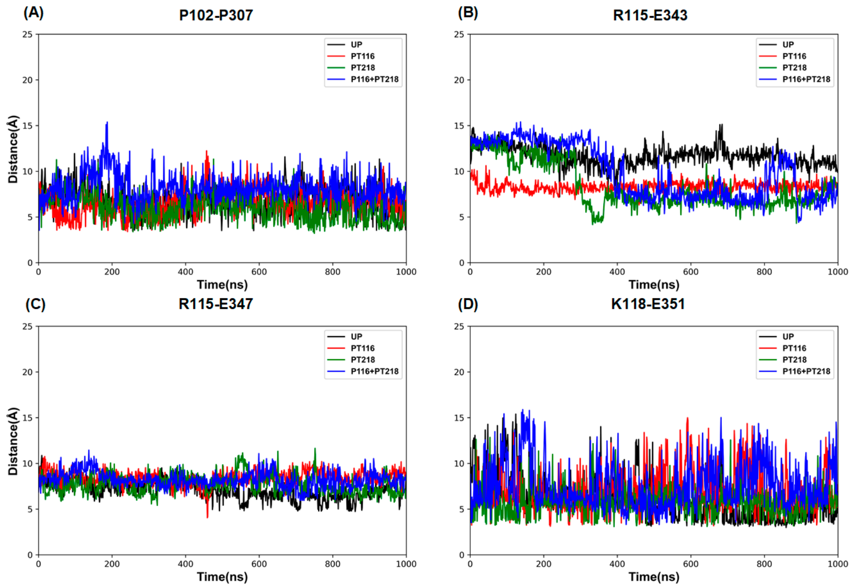

Figure S3. Time evolutions of the interaction distances between selected residue pairs during the 1000 ns MD simulations of FECH-Heme systems. (A) the distance between P102 and P107, (B) the interaction distance between R115 and E343 (C) the interaction distance between R115 and E347, (D) the interaction distance between K118 and E351.

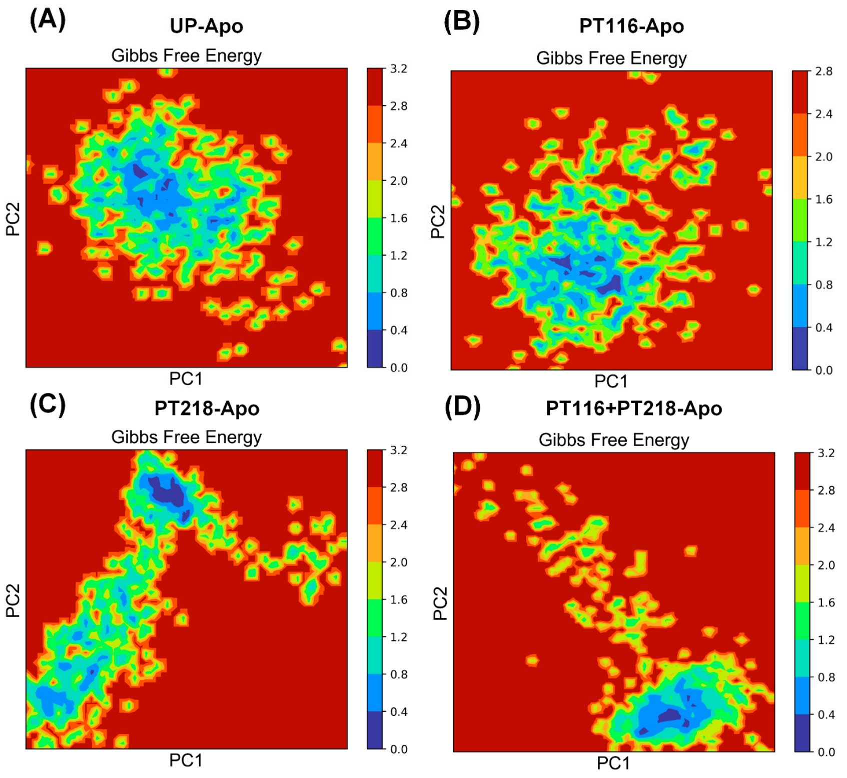

Figure S4. FELs generated by projecting the principal components, PC1, and PC2 of FECH-Apo systems in four phosphorylation states are shown in (A)-(D).

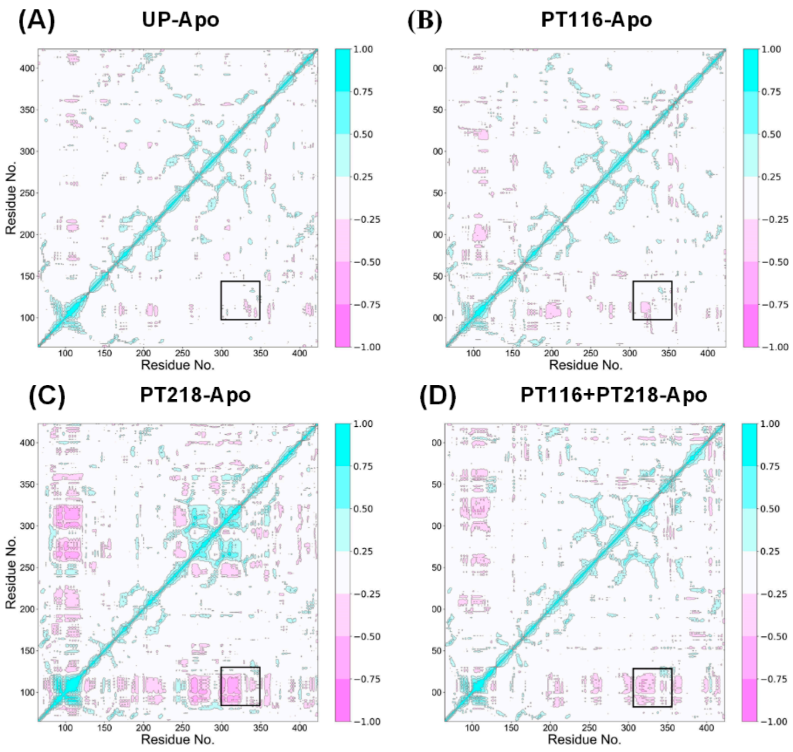

Figure S5 Dynamic cross-correlation maps of the fluctuations of the coordinates for Cα atoms of the FECH-Apo systems in four phosphorylation states are shown in (A)-(D).

1. Maisuradze, G.G.; Liwo, A.; Scheraga, H.A. Relation between Free Energy Landscapes of Proteins and Dynamics. *J. Chem. Theory Comput.* **2010**, *6*, 583–595, doi:10.1021/ct9005745.
2. Yu, H.; Dalby, P.A. A Beginner's Guide to Molecular Dynamics Simulations and the Identification of Cross-Correlation Networks for Enzyme Engineering. In *Methods in Enzymology*; Elsevier, 2020; Vol. 643, pp. 15–49 ISBN 978-0-12-821149-6.
